# Multiple dimensions of neurological injury in a liver transplant recipient with cryptococcal meningitis

**DOI:** 10.1128/asmcr.00040-24

**Published:** 2024-12-12

**Authors:** Shmuel Shoham, Sabin Thapaliya, Robin Avery, John Baddley, Veronica Dioverti, Christine Durand, Ahmet Gurakar, Andrew Karaba, Olivia Kates, Nicholas Maragakis, Nitipong Permpalung, William Werbel, Arturo Casadevall

**Affiliations:** 1 Department of Medicine, Johns Hopkins University School of Medicine, Baltimore, Maryland, USA; 2 Department of Internal Medicine, Tribhuvan University Teaching Hospital, Kathmandu, Nepal; 3 Department of Neurology, Johns Hopkins University School of Medicine, Baltimore, Maryland, USA; 4 Department of Molecular Microbiology and Immunology, Johns Hopkins Bloomberg School of Public Health, Baltimore, Maryland, USA; Pattern Bioscience, Austin, Texas, USA

**Keywords:** *Cryptococcus neoformans*, meningitis, central nervous system, fungal, intracranial pressure, immune mechanisms

## Abstract

**Background:**

Meningitis due to *Cryptococcus neoformans* continues to cause substantial morbidity and mortality in victims of this infection. Worldwide, immunosuppression due to uncontrolled HIV disease is the main risk factor. In the United States, however, a substantial percentage of cryptococcal meningitis cases occur in people with non-HIV immune compromising conditions such as solid organ transplantation. Neurological damage in cryptococcal meningitis can be caused by direct fungal-induced injury, as a consequence of elevated intracranial pressure, and by immune-mediated injury. An understanding of these pathophysiological processes and an appreciation for the roles of specific treatments to address them are necessary for optimal care of patients with cryptococcal meningitis.

**Case Summary:**

In this report, we present a case of cryptococcal meningitis in a liver transplant recipient whose disease manifestations were due to (i) fungal invasion, (ii) elevated intracranial pressure, and (iii) an over-exuberant immune response that blossomed soon after reduction of immunosuppression and clearance of cerebrospinal fluid culture.

**Conclusion:**

This patient’s course exemplifies the multidimensional nature of neurological injury in cryptococcal meningitis and the diverse treatment strategies required to address the infection and complications.

**IMPORTANCE:**

An understanding of the pathophysiological processes that cause neurological damage in cryptococcal meningitis is critical for optimal management of patients with this infection. This case highlights three such processes so that they can be quickly diagnosed and treated before irreversible damage occurs.

## INTRODUCTION

*Cryptococcus neoformans* is a ubiquitous environmental organism, which causes fungal infections and meningitis in solid organ transplant (SOT) recipients (1–3). In cryptococcosis, the damage to central nervous system (CNS) structures is caused by fungal invasion of tissues, elevated intracranial pressure (ICP), and the host response (4–6). The damage response framework has described the relationship between host response and disease manifestations (4) and informs our understanding of cryptococcosis pathogenesis and therapy (7). In transplant-related cryptococcal meningitis (CM), the cause of the initial injury is typically skewed toward that caused by the pathogen. After initial antifungal therapy, control of elevated ICP, reduction of immunosuppression, and clearance of cerebrospinal fluid (CSF) cultures, patients may experience disease flares and neurological injury from an over-exuberant immune response. This patient’s disease course demonstrates the tumultuous clinical course of an SOT recipient with CM, whose immune and other physiological responses were modulated by fungal factors, antifungal therapies, and adjustments in anti-rejection medications. In this case, fungal invasion of tissues, elevated ICP, and the host’s response each dictated clinical manifestations and required specific management.

## CASE PRESENTATION

A 51-year-old HIV-negative man with a history of alcohol-related cirrhosis underwent deceased donor liver transplantation approximately 10 months prior to presentation. The MELD score at transplant was 30. Induction immunosuppression was with methylprednisolone 500 mg. His post-transplant course was free of complications up to this point, and renal function and neutrophil counts were normal. His presenting symptoms consisted of about 2 weeks of intermittent headache, nausea, and diarrhea. He subsequently developed vertigo, lightheadedness, chills, and subjective fevers. His medications included mycophenolate mofetil 500 mg twice daily (levels not checked), tacrolimus 3 mg/day (levels ranged from 5 to 8 ng/mL; normal 5–20), and prednisone 5 mg/day. He lived in a rural part of the Mid-Atlantic region of the United States, and his activities included chopping wood. His blood pressure was 167/98 mmHg, pulse 57 bpm, and temperature 36.9°C. He had visual hallucinations, but no focal neurological abnormalities.

**Fig 1:**
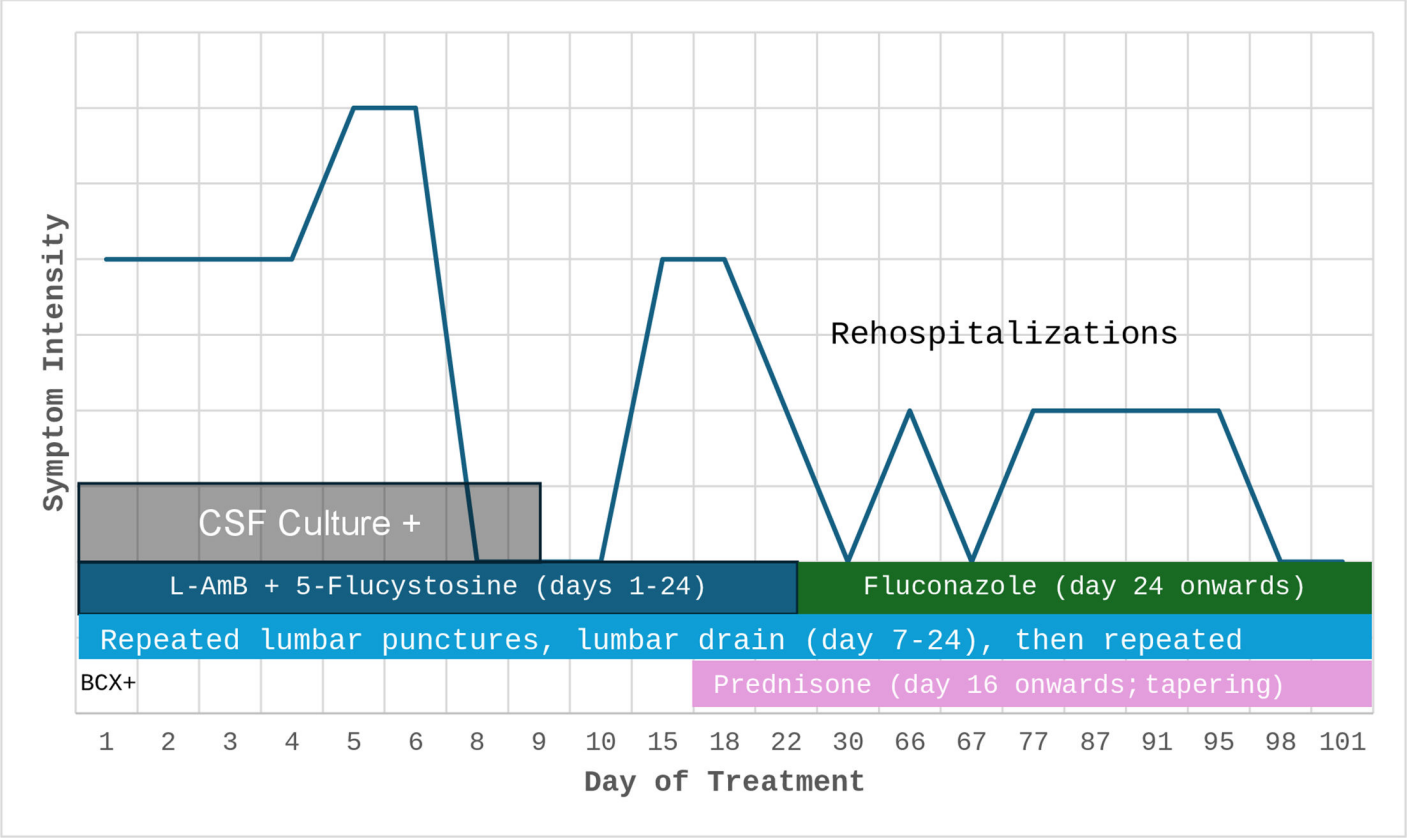
Clinical and CSF parameters. CSF=cerebrospinal fluid, L-AmB=liposomal amphotericin B, BCX=blood cultures Fevers were on days 8, 14, and 17.

Table 1 and Fig. 1 show the patient’s clinical and CSF parameters. Initial CSF testing showed a white blood cell count of 135 cells/cu mm (50% neutrophils, 35% lymphocytes, and 14% monocytes), glucose 26 mg/dL, protein 113 mg/dL, opening pressure of 42 cm H_2_O, budding yeasts on Gram and calcofluor stains, growth of *C. neoformans,* and positive cryptococcal antigen (CrAg). Serum CrAg and blood cultures obtained that same day were positive for *C. neoformans*. Blood cultures obtained 3 days later were negative.

**TABLE 1:** Clinical and CSF parameters^*a*^

Day	1	2	3	4	5	6	8	9	10	15	18	22	30	66	67	77	87	91	95	98
Symptoms	+++	+++	+++	+++	++++	++++	+	+	+	+++	+++	++	+	++	+	++	++	++	++	+
CSF parameters**^*b*^**																				
WBC (cells/mm^3^)	135	76	104	189	97	81	143			181	753	173	142	55	41	37	37	41	56	36
Protein (mg/dL)	113	100	91	87.3	105	95	83			256	447	131	82	54	49	67	73	76	61	56
Glucose (mg/dL)	26	20	24	21	28	23	24			19	25	21	65	37	37	39	39	28	44	40
Culture	+	+	+	+	+	+	+	+	-	-	-	-	-	-	-	-	-	-	-	-
CW	+	+	+	+	+	+	+	+	+	+	-	-	-	-	-	-	-	-	-	-
CrAg (CSF)**^*c*^**	+	+	+	+		+	+	+	+	+	+	+	+	+	+	+	+	+	+	+
OP (cm H_2_O)	42	43	34	41		36	LD			LD	LD	LD	23	26	22	26	27	23	23	25
Blood tests																				
BCX**^*d*^**	+																			
CrAg^*c*^	+																			
Therapy																				
AF agents^*e*^	|-Liposomal amphotericin B (L-AmB) days 1–24----------------------------------------------------------|																			
	|-Flucytosine (5-FC) days 1–23----------------------------------------------------------------------------|																			
													|-Fluconazole day 25 onward →							
Steroids										|-Prednisone day 16 onward →										
ICP control							|-Lumbar drain days 7–24, repeated CSF taps for pressure control continued afterward →													

^*a*^ Tacrolimus Day 1 = first day of treatment, CSF = cerebrospinal fluid, WBC = white blood cells, CW = calcofluor white stain, CrAg = cryptococcal antigen, OP = opening pressure, BCX = blood culture, AF = antifungal, ICP = intracranial pressure. + means present; ++, +++, and ++++ mean increasing intensity; - means absent.

^*b*^ CSF reference ranges: white blood cell count 0–5 cells/cu mm, glucose 50–75 mg/dL, and protein 15–45 mg/dL.

^*c*^ Neither the serum nor the CSF CrAg assays reported quantitative results.

^*d*^ Blood cultures were aerobic and anaerobic.

^*e*^ Flucytosine and fluconazole drug levels were not measured.

The patient was treated with liposomal amphotericin B (L-AmB) at doses ranging from 3 to 5 mg/kg/day, flucytosine 25 mg/kg four times daily, and daily lumbar punctures to control elevated ICP and neurological symptoms. Approximately 15–25 mL of CSF was removed with each procedure. Mycophenolate and prednisone were discontinued, and the tacrolimus dose was reduced. Over the first few days of therapy, he developed left facial paresis, and his mental status declined such that he required transfer to an intensive care unit (ICU). A lumbar drain was placed for ongoing ICP management, and his mental status improved dramatically. He was able to leave the ICU the next day. His CSF cell count and chemistries improved, and CSF cultures became negative after 10 days of antifungal therapy.

After approximately 2 weeks, his condition declined. He developed fever to 38.2°C, lethargy, and altered sensorium. CSF showed a white blood cell count of 753 cells (21% neutrophils; 74% lymphocytes), glucose 25 mg/dL, protein of 447.3 mg/dL, and negative cultures. Corticosteroids (initially prednisone at 40 mg/day) were started for suspicion of an immune-mediated process. His neurological status and CSF parameters improved, and the lumbar drain was removed. L-AmB and flucytosine were stopped after approximately 3 weeks, and fluconazole 800 mg/day (adjusted for renal function) was started. While in the hospital, his IgG level was found to be 373 mg/dL [reference range 610–1616 mg/dL), and he received intravenous immunoglobulin (IVIG; sucrose-free) 0.5 mg/kg. Additional immunoglobulin levels were not measured. After 41 days in the hospital, he was recommended fluconazole 400 mg/day and tapering doses of prednisone and discharged. Following this initial hospitalization, he required multiple additional hospitalizations for management of symptomatic persistently elevated ICP manifesting with symptoms that included tinnitus, headaches, nausea, and vomiting. These stabilized with CSF pressure management, and he was placed on a schedule of regular CSF taps via lumbar puncture, which he continued to receive every other week for several months.

## DISCUSSION

This case demonstrates the multidimensional pathophysiology of CM in SOT recipients. Transplant recipients are at increased risk for cryptococcosis due to their immunosuppression (8). Additional factors related to history of cirrhosis, hypogammaglobulinemia, and male gender may have also contributed (9–11).

**Fig 2:**
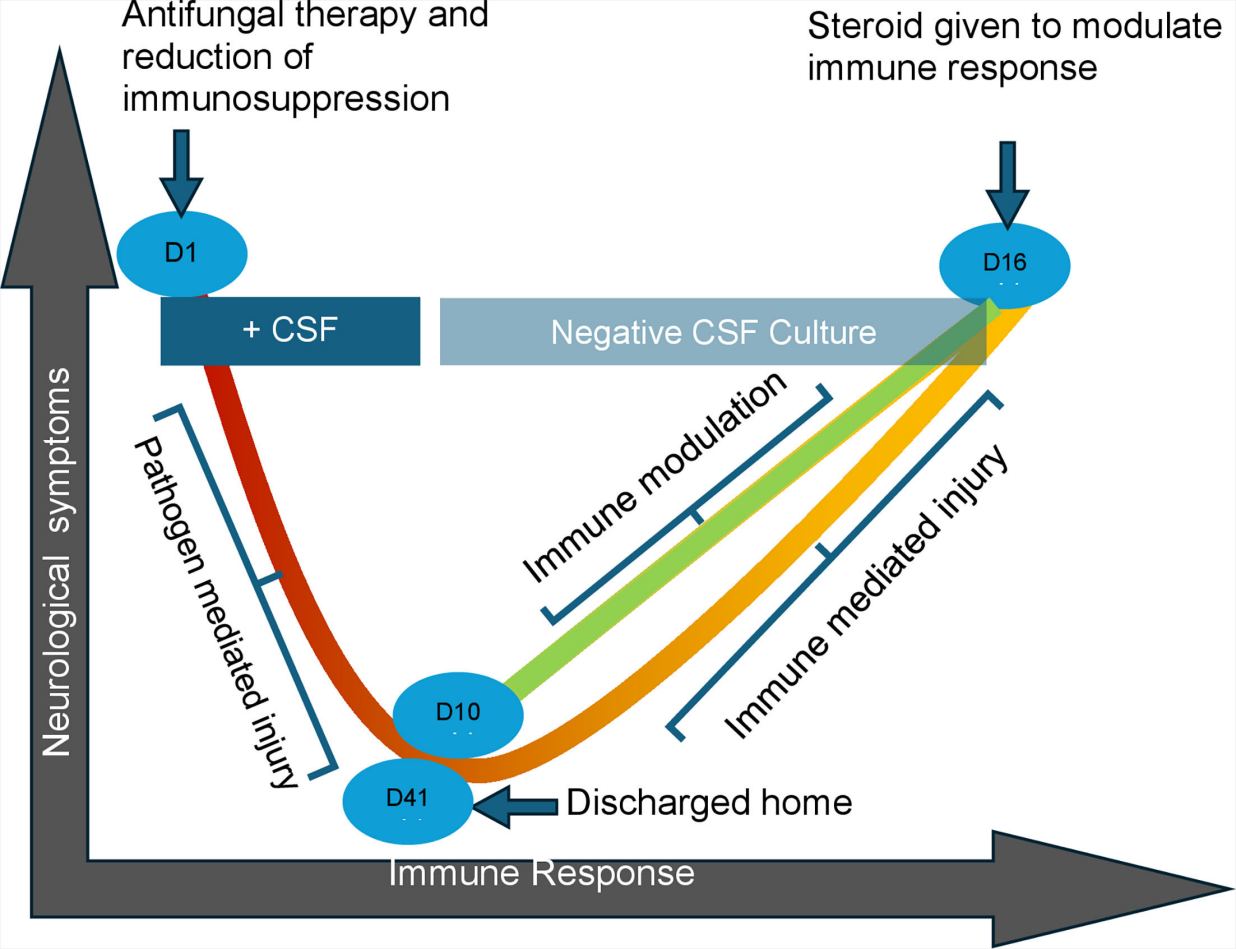
Neurological injury from cryptococcal meningitis as a function of immune status. D = day; CSF = cerebrospinal fluid. The colored lines illustrate the various phases of host/pathogen interactions. Red: disease driven by fungal invasion. Orange: disease driven by immune mediated injury. Green: disease controlled by combination of antifungal therapy and modulation of immune response. Neurological symptoms include headaches, confusion, and hallucinations. Immune response parameterers include changes in CSF pleocytosis.

According to the damage response framework, which represents host damage as a function of immune response, his initial presenting symptoms were driven by uncontrolled fungal damage in the context of an inadequate immune response (4). Treatment was with systemic antifungal therapy, ICP management, and reduction of immunosuppression to facilitate an appropriate host response to the fungus. However, he then developed symptoms due to an over-exuberant immune response. Reduction in immunosuppression can lead to immune reconstitution inflammatory syndrome (IRIS), which can occur when there is an infection, and the immune response is strengthened in HIV or other immune compromising states. IRIS can be seen following initiation of antiretroviral therapy in HIV-associated cryptococcosis (12). Additionally, with fungal damage inflicted by antifungal therapy, the immune system is exposed to previously obscured fungal antigens eliciting exuberant T-cell- and monocyte-mediated responses and clinical conditions termed paradoxical postinfectious inflammatory syndromes (PIIRS) (13). He likely had an overlap of IRIS and PIIRS. Treatment was with immune modulation using corticosteroids (14). It is possible to plot the disease stages of our patient in the damage–response parabola (Fig. 2). On admission, he was at the left side of the curve, with immunosuppression and damage coming primarily from the fungal burden, but with antifungal therapy and reduction of immunosuppressive therapy, he moved to the right side of the curve, with symptoms reminiscent of immune-reconstitution syndrome (12). Later, with initiation of corticosteroid therapy, his position moved to the left and he improved.

At the bedside, it can be challenging to ascertain whether the clinical decline is due to uncontrolled fungal growth or from an over-exuberant immune response. This is relevant as treatment with potentially toxic antifungal therapy such as higher doses of amphotericin B and immune stimulation (e.g., with interferon gamma) may be beneficial in cases of uncontrolled fungal growth, while immunosuppression with corticosteroids may be beneficial in cases of overexuberant immune response. However, treatment with corticosteroids at a time when tissue invasion and growth of *Cryptococcus* have not yet been controlled can impair the host response at a critical juncture. The ideal time for initiation of corticosteroids is not yet known, but the result of CSF cultures in the context of clinical findings can be helpful. When CSF cultures are positive, the focus should be on provision of highly effective antifungal agents. Caution should be exercised when considering intensifying immune suppression in such patients. Conversely, in patients with neurological symptoms, CrAg that remains detectable in CSF, and yeasts that are visualized in CSF by stains (e.g., Gram, calcofluor white and India ink) even after CSF culture results have turned negative, the cause of injury may be an inflammatory reaction to non-viable organisms and fungal products, and consideration should be given to initiating adjunctive corticosteroid therapy.

This patient’s course was complicated by elevated ICP, which can manifest as neurological injuries. This dimension of cryptococcal meningitis pathophysiology is driven by the cryptococcal capsule, fungal obstruction of CSF reabsorption at superior arachnoid outflow tracks, and choroiditis at the foramen of Monroe, Luschka, or Magendie (6, 15, 16). Another potential mechanism for brain swelling is cryptococcal production of mannitol, which has strong osmotic effects within the CNS (17). The cryptococcal polysaccharide can permeate the brain tissue, where it elicits neuronal swelling and can persist there for months (18–23). Elevated ICP in CM can be caused by uncontrolled fungal growth, uncontrolled inflammation, or both, or neither. As seen in this patient, elevated ICP persists after fungal clearance and modulation of inflammation and is capable of causing neurological injury independently after appropriate medical management of other disease manifestations. For some patients, this may be explained by delayed clearance of polysaccharide shed in tissues; for others, permanent scarring and impaired CSF resorption may ultimately require surgical CSF diversion for ICP control.

In summary, we present a case of cryptococcal meningitis where the interplay between (i) uncontrolled fungal growth prior to effective therapy, (ii) the host’s response to the fungus at various time points of the infection, and (iii) the neurological damage caused by elevated ICP all dictated disease manifestations and treatment approaches. More accurate tools to delineate whether clinical symptoms are caused by fungal invasion or the host’s response would be helpful to guide therapy, as would noninvasive measurements of intracranial pressure in cryptococcal meningitis.
